# Indirect Zero-Field Nuclear Magnetic Resonance Spectroscopy

**DOI:** 10.1021/acs.analchem.5c00874

**Published:** 2025-07-25

**Authors:** Kai Buckenmaier, Richard Neumann, Friedemann Bullinger, Nicolas Kempf, Pavel Povolni, Jörn Engelmann, Judith Samlow, Jan-Bernd Hövener, Klaus Scheffler, Adam Ortmeier, Markus Plaumann, Rainer Körber, Thomas Theis, Andrey N. Pravdivtsev

**Affiliations:** 1 High-Field Magnetic Resonance Center, Max Planck Institute for Biological Cybernetics, Tübingen 72076, Germany; 2 Section Biomedical Imaging, Molecular Imaging North Competence Center (MOIN CC), Department of Radiology and Neuroradiology, University Hospital Schleswig-Holstein (UKSH), Kiel University, Kiel 24118, Germany; 3 Department of Biomedical Magnetic Resonance, Eberhard-Karls University, Tübingen 72076, Germany; 4 Department of Chemistry and Physics, NC State University, Raleigh 27695, United States; 5 Institute for Molecular Biology and Medicinal Chemistry, Medical Faculty, Otto-von-Guericke-University, Magdeburg 39120, Germany; 6 Physikalisch-Technische Bundesanstalt, Berlin 10587, Germany

## Abstract

This study develops the two-field correlation spectroscopy (COSY) in zero to ultralow field (ZULF) liquid state nuclear magnetic resonance (NMR). We demonstrated the successful integration of signal amplification by reversible exchange (SABRE) hyperpolarization with two-dimensional (2D) NMR spectroscopy, enabling the detection of ZULF COSY spectra with increased sensitivity. Field cycling allowed the acquisition of two-field COSY spectra at varying magnetic field strengths, including zero-field conditions. This enabled insight into both *J*-coupling and Zeeman-dominated regimes, benefiting from ultralow field observation sensitivity and mitigating the low-frequency noise by conducting readout at higher fields (>5 μT). Our study explores the effects of polarization transfer, apodization techniques, and the potential for further application of ZULF NMR in chemical analysis exemplified for three X-nuclei and three corresponding molecules: [1-^13^C]­pyruvate, [^15^N]­acetonitrile, and [3-^19^F]­pyridine. These findings pave the way for more sensitive and cost-effective NMR spectroscopy in low-field regimes.

## Graphical abstract

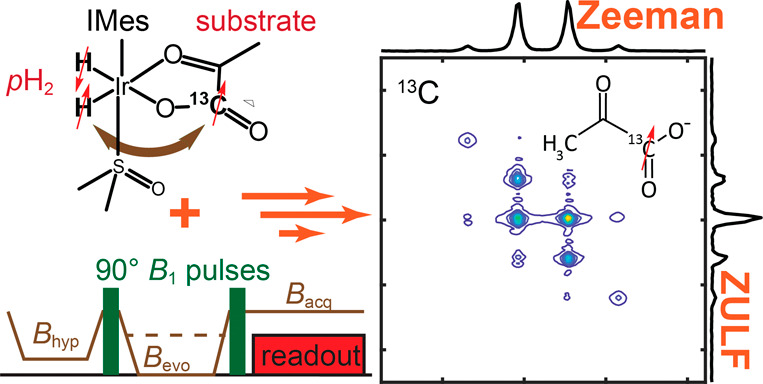

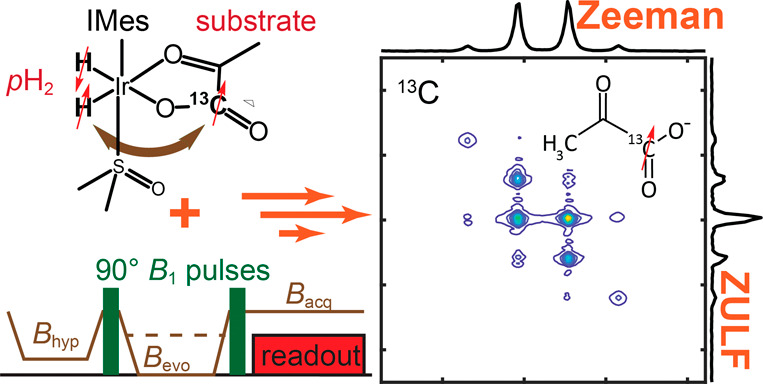

## Introduction

Nuclear magnetic resonance (NMR) is a powerful technique widely used in chemical analysis thanks to the high sensitivity of nuclear spins to their surroundings. Since its discovery in the 1970s, two-dimensional (2D) NMR spectroscopy^1^ and the subsequent development of multidimensional NMR experiments^2,3^ have significantly advanced the field, improving NMR’s ability to resolve complex spectra, leading to protein structure elucidation, and many other breakthroughs.^4,5^ Magnetic field cycling (MFC) is another technique that provides profound insights into molecular motion due to the dependence of relaxation parameters on the magnetic field.^6,7^ Inevitably, MFC and 2D NMR techniques were combined, leading to correlation spectroscopy (COSY)^8^ and total correlation spectroscopy (TOCSY)^9,10^ in two different magnetic fields. These techniques use different magnetic field strengths for signal evolution and detection, e.g., benefiting from maximum sensitivity at high fields and functional spin properties at low fields. Due to typical hardware limitations, the sample is physically moved between these different field strengths. With this technique, signal evolution even below 5 nT has been achieved.^11^

Zero and ultralow field (ZULF) NMR is gaining attraction due to its spin–spin interaction regime and the capabilities that the low-frequency ZULF regime offers (Figure 1a,b),^12–71^ such as NMR spectroscopy through metals,^18^ chemical analysis,^14,19–22^ ZULF-TOCSY,^10^ and ZULF-COSY.^23,24^ However, these advancements are impossible with Faraday-based detection coils, commonly used in high-field NMR due to their low sensitivity at low frequencies.^25^ Instead, ZULF apparatuses (Figure 1c) use either superconducting quantum interference devices (SQUIDs)^26,27^ or optically pumped magnetometers (OPMs)^28^ to detect the NMR signal. OPMs have the advantage of not requiring cryogenics and directly observing NMR signals. In contrast, SQUIDs have a much wider bandwidth than OPMs, usually limited by the electronics driving the SQUID to the 1–10 MHz range for commercially available SQUID electronics. They also show generally lower intrinsic noise levels than OPMs.^29–31^ Crucially, both detectors measure magnetic flux independent of frequency, making them ideal for quantitative ZULF NMR investigations,^26,31^ in contrast to Faraday coils in high-field MR, which measure the time derivative of the magnetic field. However, due to the intrinsic low polarization level of the sample at ZULF, usually, the sample also needs to be premagnetized^31,32^ or hyperpolarized.^26,33,34^

**Figure 1:**
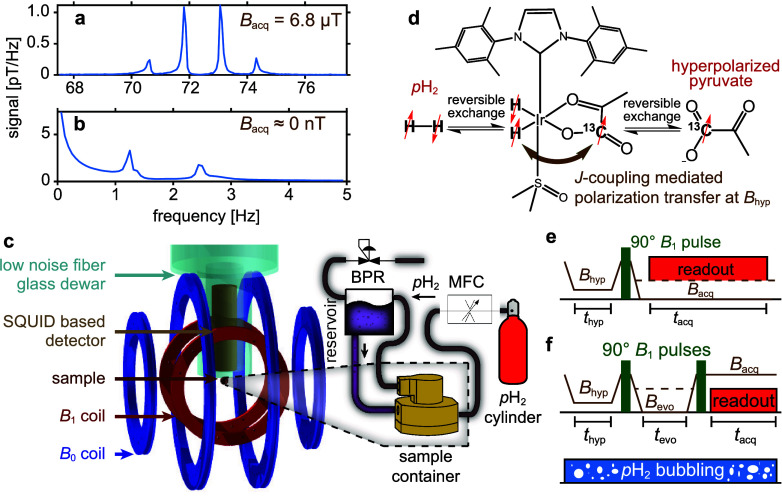
Experimental overview of SABRE at ZULF.^13^C phase-corrected NMR spectrum of [1-^13^C]­pyruvate at *B*_acq_ = 6.8 μT (a) and *B*_acq_ < 2 nT (b). Experimental ZULF NMR and SABRE setup (described in the Methods section) containing the *p*H_2_ cylinder, the mass flow controller (MFC), sample container, the reservoir, and the back-pressure regulator (BPR) (c). SABRE schematic, where *p*H_2_ and [1-^13^C]­pyruvate exchange with an iridium complex (d). At appropriate conditions (exposed to specific static magnetic fields or radiofrequency magnetic fields *B*_hyp_), polarization transfer from *p*H_2_ to ^13^C occurs.^53,54^ For example, the target heteronuclei can be polarized when the Larmor frequency difference between the *p*H_2_-derived hydride ligands and the heteronuclei is of similar order to the combination of *J*-coupling interactions: this experiment is referred to as SABRE in shield enables alignment transfer to heteronuclei (SABRE-SHEATH).^55^ Schematics of 1D ZULF (e) and ZULF COSY (f) sequences. The 1D ZULF sequence allows the measurement of the 1D spectrum at the field *B*_acq_, which can be in the ZULF regime. ZULF COSY enables the measurement of 2D correlation spectra with an evolution field of *B*_evo_ and the readout at *B*_acq_; *B*_evo_ can be in the ZULF regime.

Thanks to hyperpolarization techniques, conventional NMR is now accessible at lower target spin concentrations, reaching sub-μM sensitivity at 14 T^35^ or sub-mM sensitivity on 1 T benchtop spectrometers.^36^ Such incredible NMR sensitivity is achieved by hyperpolarization, which temporarily increases the nuclear spin polarization of selected spins far beyond their thermal, Boltzmann-determined equilibrium, thus enhancing the signal of nuclear spins. The most commonly used hyperpolarization techniques include spin-exchange optical pumping (SEOP),^37,38^ dissolution dynamic nuclear polarization (dDNP),^14^ Overhauser dynamic nuclear polarization (ODNP),^3,26,39^ and parahydrogen (*p*H_2_)-based polarization techniques including the hydrogenative parahydrogen-induced polarization (PHIP),^40^ and the non-hydrogenative signal amplification by reversible exchange (SABRE, Figure 1d).^41–43^ PHIP and SABRE are relatively inexpensive and straightforward compared to commercially available dDNP with small-footprint cryogen-free polarizers,^44–46^ and can, for example, be placed within the bore of the MRI system.^47^ In addition, SABRE provides stable hyperpolarization replenishment, which is ideal for 2D NMR at the expense of catalyst presence and polarization transfer efficiency dependence on specific substrates.^48^

## Methods

### Setup and Reactor

The experimental setup was housed within a three-layered shielding chamber, maintaining a residual magnetic field of approximately 10 nT. Consequently, magnetic field shimming was necessary to acquire ZULF NMR.

The zero and ultralow field setup (Figure 1c) consisted of a SQUID-based magnetic flux detector coupled to a second-order gradiometer. The sensor was placed within a low-noise fiberglass dewar under which the sample reactor was positioned with a hot-to-cold distance of about 13 mm. A tetra coil was used to alternate the magnetic field between hyperpolarization, *B*_hyp_, evolution, *B*_evo_, and acquisition, *B*_acq_, fields. The *B*_1_ RF field was generated with a Helmholtz coil. Directly on the *B*_1_ coil was an additional shimming coil. This previously described setup was additionally equipped with a mass flow controller and back-pressure regulator (P-7XX, IDEX) to enable experiments at pressures up to 10 bar of pH2.^26^

### *p*H_2_ Generator

Normal dihydrogen consists of approximately 25% *p*H_2_ and 75% orthohydrogen (*o*H_2_). The degree of *p*H_2_ enrichment is temperature-dependent, with enrichment levels ranging from 50% at 77 K to over 99% below 25 K. Our *p*H_2_ generator operates at cryogenic temperatures, where a paramagnetic catalyst, iron hydroxide FeO­(OH), converts normal dihydrogen gas into *p*H_2_-enriched gas. We achieved about 95% *p*H_2_ fraction using a commercially available system (XEUS-technologies).^49^ We applied a cooling temperature of 25 K, slightly above the boiling point at ambient hydrogen pressure of 20 K. The *p*H_2_ was stored in aluminum gas cylinders at a pressure of approximately 30 bar before usage.

### Sample Preparation

#### Pyruvate Sample

The sample consisted of 50 mM sodium [1-^13^C]­pyruvate (Sigma-Aldrich, article no. 490709), 5 mM [Ir­(COD)­(IMes)­Cl] precatalyst (COD = 1,5-cyclooctadiene, IMes = 1,3-bis­(2,4,6-trimethylphenyl)-1,3-dihydro-2H-imidazol-2-ylidene) (synthesized according to ref 50, with a molar mass of 640.28 g/mol), and 18 mM DMSO (Thermo Fisher Scientific, article no. 348445000), all dissolved in 7 mL of methanol (Carl Roth, Rotisolv ≥ 99.9%, article no. T909.1).

#### Acetonitrile Sample

The sample consisted of 54 mM [^15^N]­acetonitrile (Sigma-Aldrich, article no. 487864) and 0.33 mM [Ir­(COD)­(IMes)­Cl] precatalyst, also dissolved in 7 mL of methanol.

#### Fluoropyridine Sample

The sample consisted of 23 mM [3-^19^F]­pyridine (Thermo Fisher Scientific, article no. 188300050) with 1.1 mM [Ir­(COD)­(IMes)­Cl] precatalyst, dissolved in 7 mL of methanol.

### SABRE-SHEATH

Before and during NMR measurements, *p*H_2_ was bubbled into the 5 °C cold reaction chamber at ambient pressure, with a *p*H_2_ flow rate of 2 SL/h (standard liter per hour) for the [1-^13^C]­pyruvate and [3-^19^F]­pyridine samples. For the [^15^N]­acetonitrile ZULF-COSY measurement, bubbling and measurement were performed at 2.5 °C, with a *p*H_2_ flow rate of 14 SL/h and a pressure of 8 bar. During the experiments, the sample concentrations gradually changed due to the evaporation of methanol.

#### Apodization

The sine-bell apodization function in the form:

$$\mathrm{a̅}(t)=\frac{a(t)}{\max(a(t))}$$ 1

with $a(t)=\sin\left(\frac{t}{T}\pi \right)\exp\left(-\frac{t}{T/k}\right)$ , $k=\frac{T}{T_{2}}$ , where *T* is a total measurement time, and *T*_2_ is the transversal relaxation time, proved to be an excellent choice for improving the quality of ZULF COSY spectra (detailed in Section S3). Applying such a function increased SNR by a factor of 2 and reduced ringing artifacts (see effect of apodization function on ZULF COSY in the Section S5). The parameter *k* = 4, 6, and 4 for [1-^13^C]­pyruvate, [3-^19^F]­pyridine, and [^15^N]­acetonitrile was used, respectively.

### Simulations of COSY Spectra

For the simulation, the moin-spin-library^51^ (version and scripts used are available in Supporting Information) was utilized to generate the theoretical spectra. The resolution, *B*_acq_, *B*_evo_, bandwidth, and population of spin operators were selected to match those of the experimental setup.

The simulations included the first 90° pulse, the spin evolution between the 90° pulses, the second 90° pulse, and the acquisition of the resulting FID, but not the hyperpolarization phase before and during the COSY experiment. As SABRE-SHEATH also hyperpolarizes states that the product of longitudinal spin operators can represent, we also had to consider those for the initial state before the first 90° pulse. So, the initial state of the *A*_3_ *X* system, which were [1-^13^C]­pyruvate (*A* = ^1^H, *X* = ^13^C) and [^15^N]­acetonitrile (*A* = ^1^H, *X* = ^15^N) was given by the following density matrix:

$$\mathrm{\rho ̂}=\frac{1̂}{16}+p_{X}{Î}_{z}^{X}+p_{A}({Î}_{z}^{A_{1}}+{Î}_{z}^{A_{2}}+{Î}_{z}^{A_{3}})+p_{2z}{Î}_{z}^{X}({Î}_{z}^{A_{1}}+{Î}_{z}^{A_{2}}+{Î}_{z}^{A_{3}})+p_{3z}{Î}_{z}^{X}({Î}_{z}^{A_{1}}{Î}_{z}^{A_{2}}+{Î}_{z}^{A_{2}}{Î}_{z}^{A_{3}}+{Î}_{z}^{A_{1}}{Î}_{z}^{A_{3}})$$ 2

Coefficients *p* correlate with the polarization values.^52^ Furthermore, we assumed imperfect 90° pulses and chose flip angles such that the simulation data aligned with the experimental data. This compensated for the spin evolution and hyperpolarization build-up at *B*_evo_ in the experiment. The polarization’s initial values and other parameters are detailed in Table S3. When the FIDs were obtained, the simulated signal was processed like experimental spectra, including apodization.

## Results

We measured the zero-field NMR spectra directly (1D ZULF sequence, Figure 1e) and indirectly with the two-field MFC technique (ZULF COSY sequence, Figure 1f), using SQUID-based NMR and continuous SABRE in shield enables alignment transfer to heteronuclei (SABRE-SHEATH)^55^ hyperpolarization. *p*H_2_ required for SABRE-SHEATH was continuously bubbled through the sample in all our experiments. In high-field NMR, such continuous bubbling would typically result in severe susceptibility artifacts; however, not in the ZULF regime. The two-field MFC approach takes advantage of the higher NMR sensitivity at higher frequencies since the noise level at ZULF is greatly increased in the low-frequency range due to 1/*f* noise, with *f* being the frequency. The experiments with [1-^13^C]­pyruvate are detailed below, while experiments with [3-^19^F]­pyridine and [^15^N]­acetonitrile are presented in the supporting materials (Supporting Information). For signal observation, a home-built SQUID-based MFC NMR setup was used (Figure 1c, see description in methods).^26^

In the following, we introduce and compare 1D ZULF NMR spectroscopy and the two-field COSY NMR methods. The experimental data is compared to corresponding simulations.

### 1D ZULF NMR Spectroscopy

Before each readout phase, the sample was hyperpolarized using the SABRE-SHEATH technique. This was done by reducing the magnetic field for the time *t*_hyp_ to the hyperpolarization field *B*_hyp_. The highest signal of the X-nuclei *B*_hyp_ field was determined before for each substrate (see Table S1 and Section S1).

To acquire 1D ZULF spectra (Figure 1e andTable S2), the magnetic field was first increased to *B*_0_ > 10 μT, where a 90° *B*_1_ pulse in resonance with protons and the X-nucleus was applied. After that, the field was reduced to the data acquisition field, *B*_acq_, for the acquisition of free induction decay (FID) during *t*_acq_ period. The signals’ Fourier transformation and phase correction led to the 1D spectrum.

To anticipate the results from the two-field COSY experiment, we measured reference 1D NMR spectra. The FID recorded at *B*_acq_ = 6.8 μT, in the Zeeman regime, i.e., when *J*-interaction between protons and X-nuclei was much smaller than the difference of their Larmor precession frequencies, showed the expected quartet with peaks separated by the *J*-coupling frequency of ^3^ *J*_CH_ = 1.25 Hz at the ^13^C Larmor frequency for [1-^13^C]­pyruvate (Figure 1a). In contrast, the FID recorded at *B*_acq_ < 2 nT in the ZULF regime, i.e., when *J*-couplings exceeded Zeeman interactions, resulted in a spectrum with peaks at 0, *J*, and 2*J*, all corresponding to the same *J*-coupling frequency of ^3^ *J*_CH_ = 1.25 Hz (Figure 1b). The zero-field spectrum was also used as a control to confirm the absence of residual magnetic field components that could affect the ZULF COSY spectra presented below. The spectra shown here agreed with other ZULF spectra of [1-^13^C]­pyruvate found in the literature.^14,33,34^

### Two-Field 2D COSY NMR Spectroscopy

A modified COSY readout scheme was used to acquire the ZULF COSY spectra (Figure 1f). Unlike conventional COSY sequences, where the magnetic field *B*_0_ remains constant during encoding and acquisition,^3,48^ this modified approach lowers the *B*_0_ field to *B*_evo_ during the evolution period of length *t*_evo_ between the two 90° *B*_1_ pulses. For the readout phase, which lasted *t*_acq_, the magnetic field was increased to *B*_acq_. Both *B*_1_ pulses were applied at *B*_acq_. *B*_acq_ was chosen to ensure that noise bands did not obscure any NMR signals. Consequent variation of the evolution time *t*_evo_ (indirect dimension) and *t*_acq_ (direct dimension), led to 2D NMR spectra. By applying an apodization function, performing a 2D Fourier transform, and taking the absolute value (ref 56), the 2D NMR spectra were obtained (see Methods section and Section S3).

The required time for the *B*_0_ field ramps within the sequences was *t*_ramp_ = 10 ms. It is critical that the ramp field profile is consistent during the 2D experiment for each *t*_evo_.

In the following, we compare two-field COSY spectra of [1-^13^C]­pyruvate measured in the *J*-coupling regime (*B*_evo_ < 2 nT, *J*-coupling much larger than Zeeman splitting), intermediate coupling regime (*B*_evo_ = 25 nT, *J*-coupling and Zeeman splitting are of the same order of magnitude) and Zeeman regime (*B*_evo_ = 494 nT, Zeeman splitting much larger than *J*-coupling). The readout field was always the same and equal to *B*_acq_ = 6.8 μT, resulting in resonance frequencies of 72.4 and 288.3 Hz for ^13^C and ^1^H, respectively, in the direct dimension.

### Two-Field COSY of [1-^13^C]­pyruvate: *J*-Coupling Regime, *B*_evo_ < 2 nT

The indirect dimension projection of the two-field COSY experiment with *B*_evo_ < 2 nT (Figure 2 and Table S2) revealed a well-resolved spectrum with peaks at 0, ±*J*, and ±2*J* frequencies, as anticipated from the 1D ZULF NMR (Figure 1b). In the direct dimension projection, we observed the anticipated quartet peaks of [1-^13^C], separated by the *J*-coupling frequency ^3^ *J*_CH_ = 1.25 Hz, and the expected doublet of ^1^H, also split by the *J*-coupling frequency. The peak positions in both the measured and simulated data showed excellent agreement (Figure 2). By adjusting the simulation parameters (Table S3), the peak intensities were also well-matched, and all features of the experimental data were successfully reproduced in the simulations.

**Figure 2:**
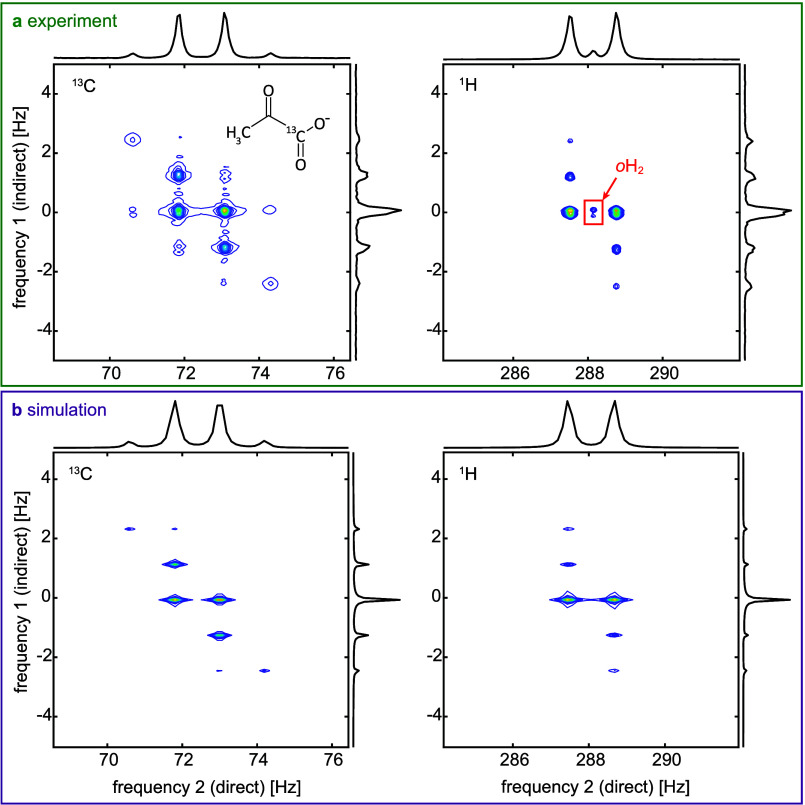
Two-field COSY spectra of [1-^13^C]­pyruvate at *B*_evo_< 2 nT and *B*_acq_ = 6.8 μT. Experimental (a) and simulated (b) absolute spectra of the ^13^C signal (left) and ^1^H signal (right) are shown together with projections in direct and indirect dimensions. We see peaks at the typical quartet positions of ^13^C and doublet positions of ^1^H in the direct frequency dimension projection and at 0, ±*J*, and ±2*J* frequencies in the indirect frequency dimension projection.

It is important to note that in the measured ^1^H ZULF COSY spectrum, an additional peak appeared at the center of the pattern (Figure 2, highlighted red box). This peak is due to *o*H_2_, a byproduct of the hyperpolarization reaction.^57,58^ It is clearly identified as *o*H_2_ since no cross-peaks to pyruvate were observed. In the simulations, only [1-^13^C]­pyruvate was considered, which explains the absence of this peak in the simulated spectra.

Note that for measuring two-fields of COSY within the *J*-coupling-dominated regime, the residual field during the evolution period must be below ≈2 nT. Even though, the whole SQUID-based NMR setup was situated within a three-layered shielding chamber (two layers of mu-metal, shielding DC fields and one layer of aluminum for RF shielding), it was necessary to use additional zeroth-order shimming coils.

### Two-Field COSY of [1-^13^C]­pyruvate: Intermediate Coupling Regime, *B*_evo_ = 25 nT

The evolution at an intermediate field of *B*_evo_ = 25 nT (Figure 3) resulted in a more complex spectrum compared to both ZULF and high-field conditions, as it represents a transition regime between the two. The peaks in the indirect dimension remained close to 0 Hz and corresponding *J* and 2*J* frequencies, but no distinct or easily interpretable pattern emerged (Figure 3a). Similar to the ZULF COSY spectra at *B*_evo_ = <2nT, the experimental peak positions at *B*_evo_ = 25 nT showed excellent agreement with simulations.

**Figure 3:**
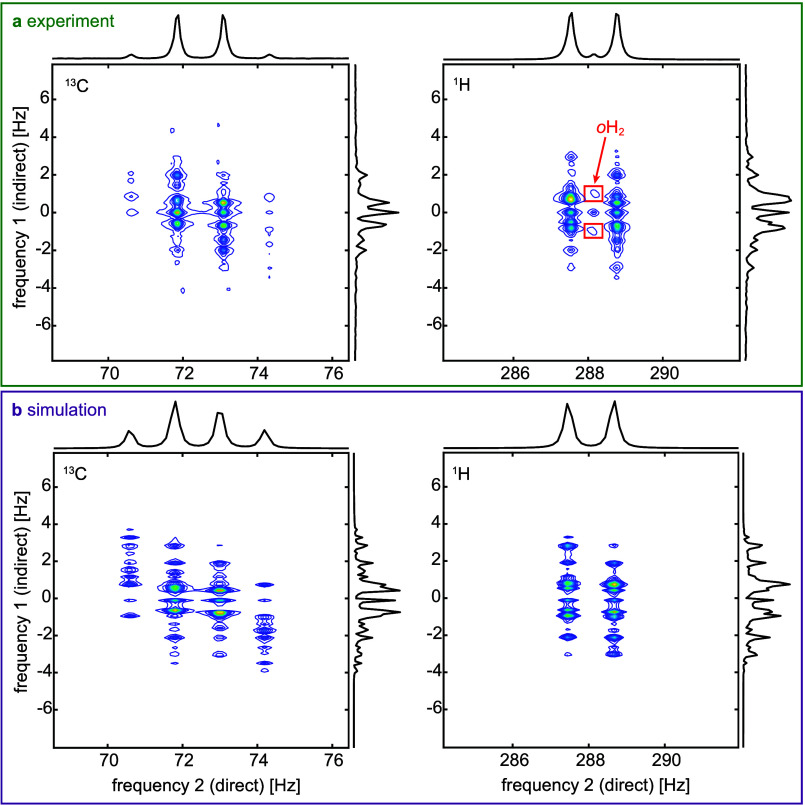
Two-field COSY spectra of [1-^13^C]­pyruvate at *B*_evo_ = 25 nT and *B*_acq_ = 6.8 μT. Experimental (a) and simulated (b) absolute spectra of the ^13^C signal (left) and ^1^H signal (right) are shown together with projections in direct and indirect dimensions. We see peaks at the typical quartet positions of ^13^C and doublet positions of ^1^H in the direct dimension. At the same time, no clear pattern can be deduced in the intermediate coupling regime in the indirect frequency dimension projection.

In the direct dimension projection, the expected quartet peaks of ^13^C, separated by the *J*-coupling frequency ^3^ *J*_CH_ = 1.25 Hz, along with the doublet of ^1^H, also split by the *J*-coupling frequency, can be observed. For *B*_evo_ = 25 nT, as expected, the *o*H_2_ peak appeared at around 1 Hz (the proton Larmor frequency at *B*_evo_) in the indirect dimension, placed between the proton’s doublet in the direct dimension (Figure 3a, red boxes). An additional peak at 0 Hz was also observed in the indirect dimension, which is explained by the production of hyperpolarization during *t*_evo_ at *B*_evo_. This phenomenon is further discussed below.

### Two-Field COSY of [1-^13^C]­pyruvate: Zeeman Regime, *B*_evo_ = 494 nT

The magnetic field strength of the evolution field *B*_evo_ = 494 nT falls within a range where the two-field COSY spectrum displays Zeeman-dominated interactions. In this regime, the 2D spectrum exhibited symmetrical patterns, and the peaks can be attributed to auto- and cross-peaks like in a classical high-field heteronuclear COSY spectrum of [1-^13^C]­pyruvate (Figure 4 and Table S2), with the difference that now both nuclei are measured simultaneously. Both projections of the spectrum on the direct and indirect frequency dimension revealed the anticipated quartet peaks at the ^13^C Larmor frequency and doublet structure for ^1^H with the *J*-coupling frequency ^3^ *J*_CH_ = 1.25 Hz.

**Figure 4:**
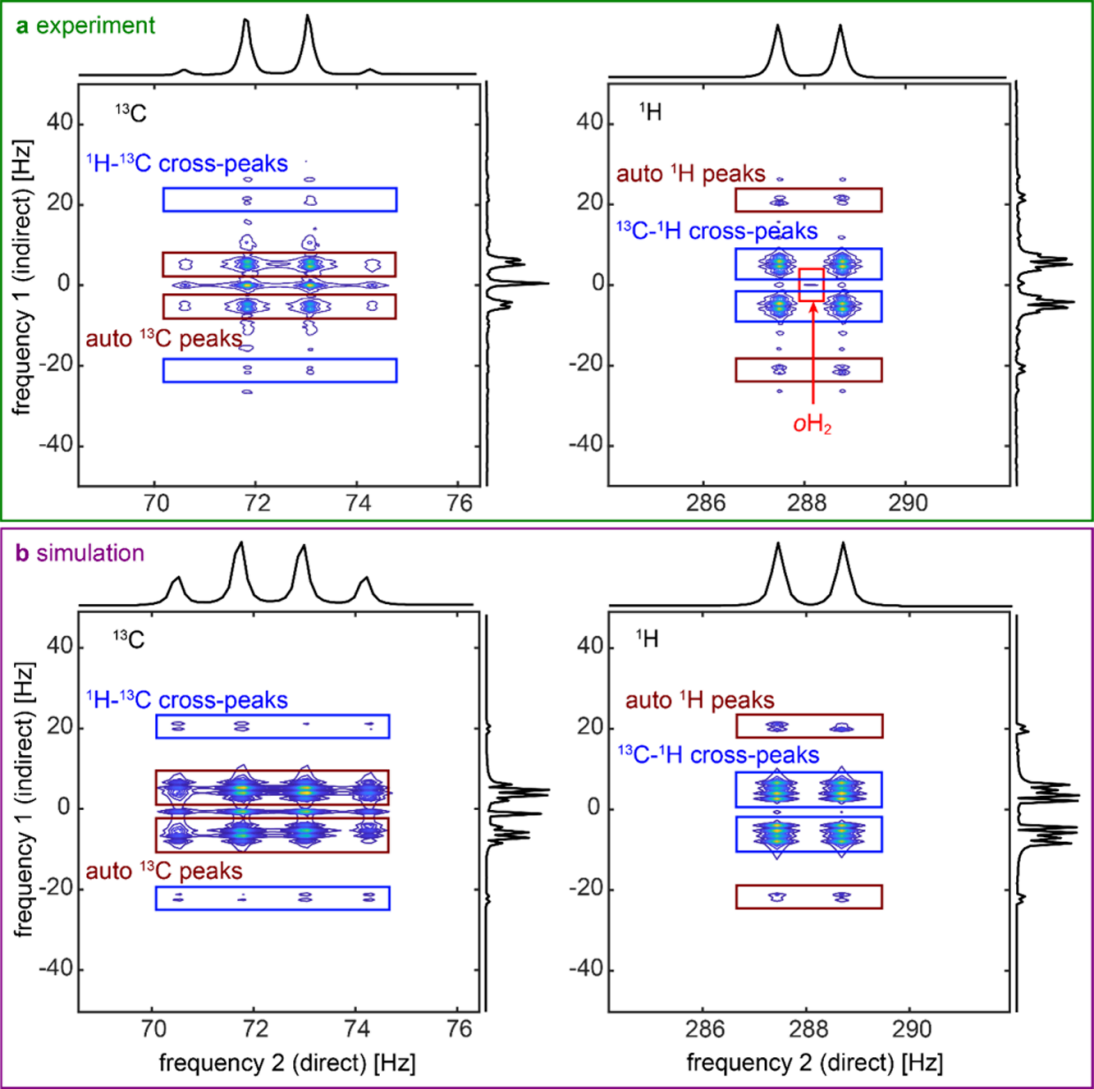
Two-field COSY spectra of [1-^13^C]­pyruvate at *B*_evo_ = 494 nT and *B*_acq_ = 6.8 μT. Experimental (a) and simulated (b) absolute spectra of the ^13^C signal (left) and ^1^H signal (right) are shown together with projections in direct and indirect dimensions. We see peaks at the typical quartet positions of ^13^C and doublet positions of ^1^H in the direct and indirect dimensions. Indirect projections additionally comprise ^1^H–^13^C and ^13^C–^1^H cross-peaks. The order of nuclei in cross-peak assignment reflects the order of indirect and direct encoding.

Note that the ^1^H signal was significantly lower than that of ^13^C. As a result, the ^13^C–^1^H cross-peak at the directly observed ^1^H signal exhibits greater intensity than the ^1^H auto correlation peak. This discrepancy arises because ^13^C is predominantly hyperpolarized via SABRE-SHEATH. A different pattern would emerge if the *B*_hyp_ field was selected such that the ^1^H pyruvate nuclei are primarily polarized.

An unexpected feature of this measurement was the presence of prominent peaks at 0 Hz in the indirect dimension of the experimental ^13^C signal (Figure 4a). During the evolution time between the two 90° pulses, *B*_evo_ was set to 494 nT, which was close to the optimal hyperpolarization field *B*_hyp_ of 350 nT. As a result, longitudinal polarization builds up during this period. It is subsequently excited by the second 90° pulse in the two-field COSY sequence, resulting in peaks at 0 Hz in the indirect direction.

## Discussion

In a previously published two-field NMR study containing a ZULF evolution field, the correlation between quadrupolar frequencies in a spin-1 system was measured for polycrystalline solids.^59^ However, the frequency range of the spectra was at ≈100 kHz, in contrast to the 2D spectra demonstrated here with *J*-coupling signals at around 0 Hz.

Combining hyperpolarized samples with the SQUID-based NMR system allowed the acquisition of ZULF-COSY spectra within a few hours. Replacing the SQUID-based magnetic field detector with a conventional Faraday detection coil would also be possible due to the enormous hyperpolarized signal intensities, further simplifying the system. This approach allowed the resolution of *J*-coupling constants in the Hz range without needing a superconducting magnet. In contrast, the experiment described in ref 11, which used a 9.4 T high-resolution NMR with a shuttle system to reach the ZULF regime, required about 60 h to acquire COSY spectra, resolving *J*-coupling constants only in the 100 Hz range and with some residual field of ∼100 nT.

1/*f* noise, especially below 100 Hz, often limits the signal-to-noise ratio (SNR) of ZULF NMR systems. This noise can originate from various sources, including the intrinsic noise of the magnetic field detector itself,^25,60^ noise from system components such as DC and AC power sources that drive the magnetic field coils, or unshielded external noise sources. The two-field COSY approach allows the acquisition of ZULF spectra at higher acquisition fields, offering the advantage of shifting the acquisition bandwidth to a region with lower intrinsic noise levels for NMR systems limited by 1/*f* noise at low frequencies. This is an advantage over the method presented in ref 23, where ZULF COSY was performed without MFC at zero field. However, the MFC ZULF-COSY approach has the trade-off of longer acquisition times than conventional 1D NMR spectra (Table S2). The 1D ZULF spectra (Figure 1) were acquired in less than 2 min with a total acquisition time of 16 s resulting in a resolution of 62.5 mHz (4 transients were averaged). The 2D ZULF COSY was measured in more than 1 h resulting in a slightly smaller resolution depending on the maximum value of the sweep variable *t*_evo_ (no averaging was used). This indicates that a very similar spectral resolution was achieved in both cases. Potentially, the longer acquisition time could be mitigated using ultrafast 2D techniques.^61^

It is important to note that for two-field COSY spectra with evolution time *t*_evo_ close to zero-field conditions (<100 nT), the bandwidth of the spectra is primarily determined by the *J*-coupling constant of the investigated substrate. This small bandwidth allows much faster acquisition of two-field COSY spectra compared to conventional COSY spectra. While the bandwidth of traditional COSY spectra can be reduced by folding higher frequencies into the bandwidth of the indirectly measured frequency dimension, the acquisition times remain significantly longer.^48^

Due to the low SNR at low frequencies without postprocessing filters, as discussed above, evaluation was challenging. However, the apodization function transformed difficult-to-analyze data into spectra with reduced noise (by a factor of ≈2) presented above. More effort into the measurements and noise reduction would have been needed without the apodization function (detailed in Section S3).

At zero magnetic field, the spins do not precess, and therefore the macroscopic magnetization does not precess in the plane perpendicular to *B*_0_ as in conventional NMR. Instead, the spins oscillate between different spin states along a single axis (one-dimensional oscillation).^33,34^ Therefore, it was critical to correctly calibrate the phase of the 90° pulse to acquire 1D ZULF spectra so that the axis of these one-dimensional oscillations was perpendicular to the pick-up coil (Figure 1b,e). Otherwise, the signal intensity would be significantly reduced or unobservable.^33^ However, at the acquisition field, *B*_acq_, of the two-field COSY sequence (Figures 1e and 2 **–** [Fig fig4]), the spin precession is sufficiently high that no phase adjustment of the 90° pulses is required. Before conducting ZULF spectra, shimming must be employed as the shielding chamber has a low residual field. Measuring 1D ZULF NMR spectra by adjusting the shim coil currents leads to approximately 1 nT residual field. We used such experiments for shimming, and the ZULF COSY experiment took much longer to execute (>1 h) than conventional FID acquisition (≈2 min). So, it may not be reasonable to use ZULF COSY for acquiring 1D ZULF spectra if time-consuming shimming is necessary.

In the intermediate regime of the two-field COSY experiment, the peaks of [1-^13^C]­pyruvate, which were located close to 0 Hz in the indirect dimension in the ZULF regime, drift outward as the evolution field *B*_evo_ is increased. Because now both the *J*-coupling and the Zeeman interaction are prominent in the indirect dimension, a complicated spectrum showing no distinguishable pattern is obtained. In the Zeeman regime, the spectrum shows the expected conventional COSY spectrum with auto- and cross-peaks^3,48^ (Figure 4).

The fundamental findings of the above-discussed results were reproduced with another *A*_3_ *X* type system of [^15^N]­acetonitrile (Sections S4.1, S4.2, and S5). While in the ZULF regime, the spectrum pattern shows the same behavior as for the [1-^13^C]­pyruvate, only auto- and cross-peaks of the ^15^N and not the ^1^H are visible in the Zeeman regime. As the proton peaks in the ZULF COSY spectrum for the ^13^C already were low, for the [^15^N]­acetonitrile, the protons were insufficiently polarized to be observed. A ZULF COSY spectrum of [3-^19^F]­pyridine was also measured, but since the molecule is asymmetric with multiple different *J*-coupling constants, the spectrum was far too complex to discuss in much detail here (Sections S4.3 and S5).

Depending on the hyperpolarization procedure, different spin states can be populated, resulting in different quantum coherences after excitation (off-diagonal terms in the density matrix)^48^ and hence different spectra. Simulations can help to deduce the initial state of the spin system. For example, when pyruvate was hyperpolarized with dissolution dynamic nuclear polarization,^14^ the peak of the ZULF spectrum at 2*J* had half the amplitude of the peak at *J*. This is different from what was observed for ZULF spectra of pyruvate hyperpolarized with SABRE-SHEATH (Figure 1b and ref 33). For SABRE, it strongly depends on the hyperpolarization conditions *B*_hyp_, *t*_hyp_ and methods (SABRE-SHEATH,^62^ low field SABRE,^63^ alt-SABRE,^64^ LIGHT-SABRE,^65,66^ MACHETE^67^) to which degree the system is hyperpolarized and to which degree the different quantum coherences appear, resulting in different peak heights of the one- and two-dimensional NMR spectra. For the simulations, a combination of longitudinal single and products of two and three spin operators was considered to represent the initial hyperpolarization state of the system to fit the measurements (Section S6). Both the flip angle and polarization data, needed in the simulations to fit the experiment, stayed identical for different ligands in the same regime but differed between regimes. The only difference in the experiment was the evolution field *B*_evo_. So, there was no experimental indication of why the flip angles vary significantly from the expected 90° pulse. We explained this effect by not considering hyperpolarization effects during the evolution phase at different *B*_evo_. The production of hyperpolarization and relaxation at varying *B*_evo_ was different. This could be added explicitly by introducing a chemical exchange of *p*H_2_ and substrate, as proposed previously^51,68^ but was not tested here.

## Conclusions

We were able to present low field two-dimensional two-field COSY for different evolution fields *B*_evo_, molecules, and X-nuclei (^13^C, ^15^N, and ^19^F). An apodization method helped to improve the quality of the acquired spectra significantly. For *B*_evo_ < 2 nT, we could show results where only the *J* coupling interactions were the relevant variable responsible for the indirect dimensional spectrum. Combined with the high-field spectrum in the direct dimension, the two-dimensional figure showed an asymmetric peak pattern, revealing connections between different transition frequencies of the ZULF and Zeeman regimes. The result of the indirect dimension projection was the desired zero-field spectrum. Slight deviations from the simulated zero-field spectrum can indicate the magnetic field inhomogeneity or deviation from the zero field and can be used to measure the residual field. The method can become an alternative to the traditional direct measurement of zero-field NMR. Although we have used the SQUID system to measure the signal here, we envision that, using higher magnetic fields and simple Faraday coils, zero-field spectra can be measured using the two-field COSY method discussed, making ZULF spectroscopy more sensitive and accessible. However, the use of the higher field could potentially compromise the precision of the zero field, which should be well controlled.

The ZULF NMR tries to become a viable analytical technique. However, it lacks resolution and chemical specificity.^42^ A two-field method can boost the chemical specificity, making ZULF even more analytically useful.

## Supplementary Material





## Notes

Additional experimental details, apodization method, results for [^15^N]­acetonitrile and [3-^19^F]­pyridine, Figures S1–S10, and Tables S1–S3 (PDF)
Simulation code^58,69,70^ (ZIP)
