# Supplementary material for: Indirect Zero-Field Nuclear Magnetic Resonance Spectroscopy
Source: Anal Chem. 2025 Jul 25;97(32):17336–44. doi: 10.1021/acs.analchem.5c00874 (PMC12368839; doi:10.1021/acs.analchem.5c00874)
Supplement: Supplementary file 2 [file ac5c00874_si_001.pdf]

# Supporting Information for
# Indirect Zero-Field NMR Spectroscopy

Kai Buckenmaier[1,*], Richard Neumann[1], Friedemann Bullinger[1], Nicolas Kempf[1], Pavel Povolni[1], Jörn Engelmann[1], Judith Samlow[1], Jan-Bernd Hövener[2], Klaus Scheffler[1,3], Adam Ortmeier[4], Markus Plaumann[5], Rainer Körber[6], Thomas Theis[4] and Andrey N. Pravdivtsev[2]

*Corresponding author, Kai Buckenmaier kai.buckenmaier@tuebingen.mpg.de

Kai Buckenmaier[1,*], Richard Neumann[1], Friedemann Bullinger[1], Nicolas Kempf[1], Pavel Povolni[1], Jörn Engelmann[1], Judith Samlow[1], Jan-Bernd Hövener[2], Klaus Scheffler[1,3], Adam Ortmeier[4], Markus Plaumann[5], Rainer Körber[6], Thomas Theis[4] and Andrey N. Pravdivtsev[2]

[1] High-Field Magnetic Resonance Center, Max Planck Institute for Biological Cybernetics; Tübingen, 72076, Germany
[2] Section Biomedical Imaging, Molecular Imaging North Competence Center (MOIN CC), Department of Radiology and Neuroradiology, University Hospital Schleswig-Holstein (UKSH), Kiel University, 24118, Kiel, Germany
[3] Department of Biomedical Magnetic Resonance, Eberhard-Karls University; Tübingen, 72076, Germany
[4] Department of Chemistry and Physics, NC State University; Raleigh, 27695, USA
[5] Institute for Molecular Biology and Medicinal Chemistry, Medical Faculty, Otto-von-Guericke-University; Magdeburg, 39120, Germany
[6] Physikalisch-Technische Bundesanstalt; Berlin, 10587, Germany

## Contents

1. Optimal $B_{hyp}$ field ..... 2
2. Sequence parameters ..... 3
3. Apodization ..... 4
4. Two-field COSY spectra in the ZULF and Zeeman regime of [$^{15}$N]acetonitrile and [3-$^{19}$F]pyridine ..... 7
   4.1. ZULF regime ZULF COSY spectrum of [$^{15}$N]acetonitrile ..... 7
   4.2. Zeeman regime two-field COSY spectrum of [$^{15}$N]acetonitrile ..... 8
   4.3. ZULF regime ZULF COSY spectrum of [3-$^{19}$F]pyridine ..... 10
5. Effect of apodization on [$^{15}$N]acetonitrile and [3-$^{19}$F]pyridine ZULF COSY spectra ..... 11
6. Simulation parameters ..... 12

# 1. Optimal $B_{hyp}$ field

To achieve maximum enhancement under SABRE-SHEATH conditions, we used a simple FID readout sequence in which the hyperpolarization field, $B_{hyp}$, was systematically varied (see **Figure S1** and **Table S1**). As in the sequences described in the main manuscript, parahydrogen was continuously bubbled through the sample reactor. The acquired data was Fourier transformed and the spectra showing the absolute value of the X-nucleus signal were plotted as a function of $B_{hyp}$ (**Figure S2** upper row). To determine the optimal hyperpolarization field the absolute value of the X-nucleus MR signal was integrated, and the $B_{hyp}$ that produced the highest signal was identified and used for other experiments (**Figure S2** middle row, dashed red line).

**Figure S2** bottom row also shows the spectra of the $^{1}$H signal. For [1-$^{13}$C]pyruvate, the $^{1}$H enhancement correlates directly with the [1-$^{13}$C] enhancement. In contrast, for [$^{15}$N]acetonitrile and [3-$^{19}$F]pyridine, only the $^{1}$H signal of orthohydrogen (with peaks at 2200.5 Hz and 2202.0 Hz for [$^{15}$N]acetonitrile and [3-$^{19}$F]pyridine, respectively) scales with the X-nucleus signal. The proton signal of the substrate shows a different trend and signal enhancement even at $B_{hyp}$ = 0.

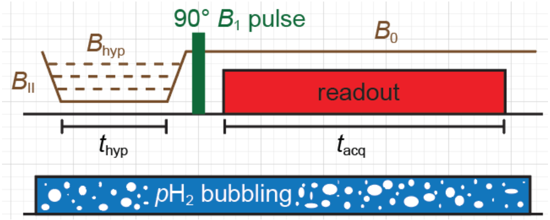


***Figure S1:*** *Schematic of the sequence used for determining optimal $B_{hyp}$ for the SABRE-SHEATH condition.*

***Table S1:*** *Sequence parameters used for determining optimal $B_{hyp}$*

| substrate | $t_{hyp}$ [s] | $t_{acq}$ [s] | $B_0$ [μT] |
|---|---|---|---|
| [1−$^{13}$C]pyruvate | 4 | 2 | 6.8 |
| [$^{15}$N]acetonitrile | 4 | 4 | 51.7 |
| [3-$^{19}$F]pyridine | 4 | 4 | 51.7 |

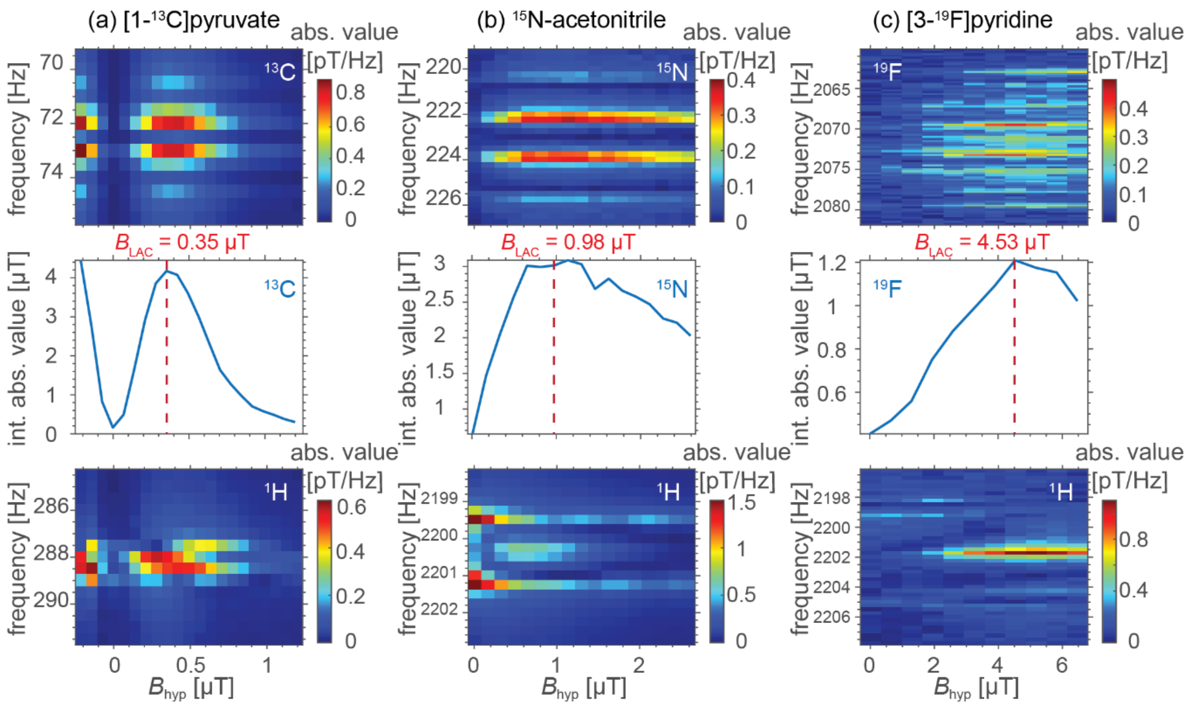


**Figure S2:** X-nucleus spectra (upper row), integrated X-nucleus MR signal (middle row), and $^{1}$H spectra (bottom row) of [1-$^{13}$C]pyruvate (**a**), [$^{15}$N]acetonitrile (**b**) and [3-$^{19}$F]pyridine (**c**).

## 2. Sequence parameters

The sequence parameters of all used sequences within the main manuscript are shown in **Table S2**.

***Table S2: Sequence parameters.*** *The table shows the sequence parameters of the discussed results.*

| Figure | $t_{hyp}$ [s] | $B_{hyp}$ [μT] | $t_{evo}$ [s] | steps | avg. | $B_{evo}$ [nT] | $t_{acq}$ [s] | $B_{acq}$ [μT] | $t_{total}$ |
|---|---|---|---|---|---|---|---|---|---|
| **ZULF FID** | | | | | | | | | |
| **1a** | 10 | 0.35 | – | – | 4 | – | 16 | 6.8 | 1min 45s |
| **1b** | 10 | 0.35 | – | – | 4 | – | 16 | < 0.002 | 1min 45s |
| **two field ZULF COSY** | | | | | | | | | |
| **2** | 8 | 0.35 | 0–15 | 151 | 1 | < 0.002 | 16 | 6.8 | 1h 2min |
| **3** | 8 | 0.35 | 0–9.4 | 151 | 1 | 25 | 16 | 6.8 | 1h 1min |
| **4** | 8 | 0.35 | 0–2.8- | 701 | 1 | 493 | 16 | 6.8 | 4h 44min |
| **S6** | 16 | 0.982 | 0–8 | 81 | 2 | < 0.002 | 8 | 51.7 | 1h 16min |
| **S7** | 16 | 4.857 | 0–6 | 281 | 2 | 1305 | 8 | 51.7 | 3h 55min |
| **S8** | 20 | 4.531 | 0–5 | 201 | 2 | < 0.002 | 8 | 51.7 | 3h 58min |

# 3. Apodization

Apodization is an NMR spectroscopy technique that improves the quality of spectral data by reducing artifacts and increasing the signal-to-noise ratio.[72] Apodization refers to the process of applying a mathematical function known as an apodization function,[73] to the time-domain signal before its Fourier transformation. For example, raw data have inherent noise and imperfections due to finite acquisition time, and sampling can lead to broadening spectral lines, reducing the signal-to-noise ratio (SNR). Apodization addresses these issues, resulting in better-defined peaks and more accurate quantification of spectral features.

For example, the NMR signal is measured only during a narrow window that can be approximated as a rectangular function defined as:

$$f(t) = f(t) = \begin{cases} 1, & |t| \leq 0.5 \\ 0, & else \end{cases} \tag{S1}$$

The Fourier transform of a rectangle function yields:

$$F(\omega) = \int_{-\frac{1}{2}}^{\frac{1}{2}} \exp(-i\omega t)\,\mathrm{d}t = \mathrm{sinc}(\omega/2). \tag{S2}$$

Consequently, the Fourier transform of a finite data set can introduce artifacts - peaks that do not exist in the ideal spectrum. The data can be preprocessed by applying apodization before the Fourier transform to reduce these windowing effects. Various apodization functions are already discussed in Ref. 73, p. 408, including a sine-bell function:

$$s(t) = \sin\left(\frac{t}{T}\pi\right), \tag{S3}$$

where $T$ is the total measurement time.

Another key reason for applying apodization is to enhance the resolution of multidimensional spectra.[1] The envelope of the FID typically follows an exponential decay function:

$$g(t) = M_0 \exp(-t/T_2), \tag{S4}$$

where $M_0$ is the initial amplitude and $T_2$ the transverse relaxation time.

The SNR can be improved by multiplying the FID spectrum with an exponential decay as an apodization function

$$h(t) = \exp\left(-\frac{t}{T/k}\right) \tag{S5}$$

with

$$k = \frac{T}{T_2} \tag{S6}$$

being the exponential weighting constant. For different $T_2$ a different $k$ must be chosen such that the apodization function matches the envelope of the FID spectrum.

Combining the two parts of the apodization, the final apodization function is

$$a(t) = \sin\left(\frac{t}{T}\pi\right)\exp\left(-\frac{t}{T/k}\right). \tag{S7}$$

To have comparable results, the apodization function is normalized.

$$\bar{a}(t) = \frac{a(t)}{\max(a(t))}, \tag{S8}$$

**Figure S3** displays this apodization function for different weighting parameters *k*. It combines the advantages of suppressing window effects with SNR improvement.

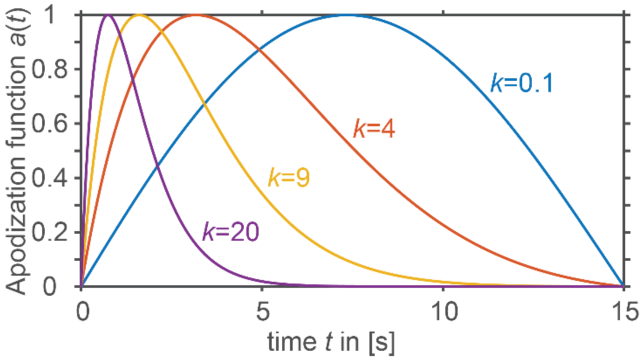


**Figure S3: Apodization functions for different weighting factors.** The figure shows the effect of the exponential weighting factor *k* on the apodization function.

The Fourier transformation of the product of the apodization function $\bar{a}(t)$ with the FID $d(t)$, yields the spectrum with applied apodization:

$$S(\omega) = \int d(t)\bar{a}(t)\exp(-i\omega t)\,dt \tag{S9}$$

Applying the apodization function (**Figure S4** middle) to the [1-$^{13}$C]pyruvate data (**Figure S4** upper row) acquired with the ZULF COSY sequence (**Figure 1f**) results in the 2D dataset with significantly reduced noise (**Figure S4** bottom). As expected, the sine bell attenuates the signal at short evolution times, while the exponential decay suppresses the signal at long evolution times. However, a reduction in amplitude of approximately a factor of $\frac{1}{2}$ is observed, even though the SNR might be increased. Therefore, the amplitude of the apodized spectra can only be given in arbitrary units (a.u.).

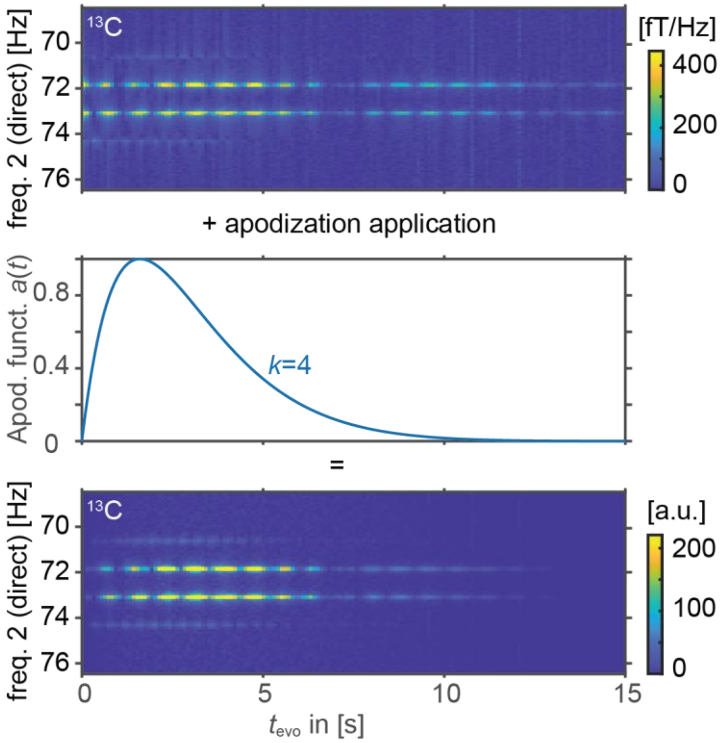


**Figure S4: Time-frequency spectrum of [1-$^{13}$C]pyruvate**. The upper figure displays the COSY spectrum with Fourier transformation applied only across the direct frequency dimension. The middle panel shows the apodization function. The bottom panel shows the product of the apodization function and the top spectrum.

Applying the Fourier transform in the indirect dimension produces the ZULF COSY spectra shown in **Figure S5**. A comparison between the ZULF COSY spectrum without apodization (**Figure S5a**) and the spectrum with apodization applied (**Figure S5b**), reveals significant improvements.

The application of apodization results in enhanced SNR by reducing the noise by an estimated factor of 2, and diminished ringing artifacts. Notably, in the projection along the indirect frequency dimension, the outer peaks of the quintuplet become clearly visible in the apodized spectrum. However, due to the modification of the measurement data by apodization, the amplitude is altered and given in a.u. (**Figure S5**).

Apodization was applied in ZULF COSY figures of the results section within the main manuscript and all subsequent ZULF COSY figures, allowing for a more refined and detailed analysis. For [1-$^{13}$C]pyruvate, $k$ = 4 ([3-$^{19}$F]pyridine, $k$ = 6 and for [$^{15}$N]acetonitrile, $k$ = 4) proofed to be an optimal.

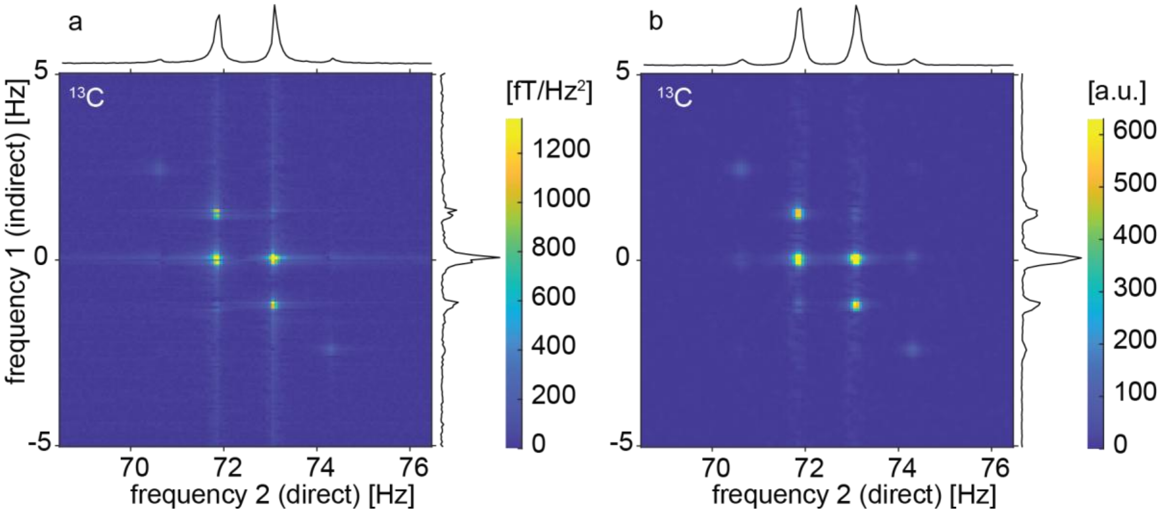


**Figure S5: Effect of apodization to the ZULF COSY spectrum of [1-$^{13}$C]pyruvate.** The figure illustrates the effect of apodization on two-dimensional spectra. The spectrum without apodization (**a**) does not display the outer peaks in the indirect dimension projection of the $^{13}$C, whereas these peaks become clearly observable in the apodized spectrum (**b**). The SNR enhancement resulting from applying apodization is apparent in both the two-dimensional spectrum and its projections.

# 4. Two-field COSY spectra in the ZULF and Zeeman regime of [$^{15}$N]acetonitrile and [3-$^{19}$F]pyridine

Additionally, to the [1-$^{13}$C]pyruvate, spectra of [$^{15}$N]acetonitrile and [3-$^{19}$F]pyridine will be shown. [$^{15}$N]acetonitrile is similar to [1-$^{13}$C]pyruvate an $\mathrm{A^3X}$ system, and thus, the results show the same pattern. [3-$^{19}$F]pyridine is a far more complex molecule, and so is the obtained spectrum.

## 4.1. ZULF regime ZULF COSY spectrum of [$^{15}$N]acetonitrile

The results of ZULF COSY [$^{15}$N]acetonitrile are as anticipated from the [1-$^{13}$C]pyruvate data as both [$^{15}$N]acetonitrile and [1-$^{13}$C]pyruvate, are $\mathrm{A^3X}$ systems.

The ZULF evolution field of $B_{\mathrm{evo}} < 2\,\mathrm{nT}$ results in a zero-field spectrum in the indirect dimension, where both the $^{15}$N and $^{1}$H projections display a well-defined quintuplet with peaks at 0, ±$J$, and ±2$J$ frequencies. Note that for [$^{15}$N]acetonitrile $^{3}J_{\mathrm{NH}}$ is about 1.75 Hz.

For this experiment again, it was necessary to use further shimming coils. The shimming process itself was performed by acquiring ZULF FID spectra while varying the shimming fields accordingly.

In the direct dimension projection, the quadruplet peaks of $^{15}\mathrm{N}$, separated by the $J$-coupling frequency, as well as the doublet of $^{1}\mathrm{H}$, also split by the $J$-coupling frequency, can be observed.

To align the simulation with experimental results, exactly the same simulation parameters as for the [1-$^{13}$C]pyruvate are selected. The parameters can be found in **Table S3**. The peak positions in the measured and simulated data show excellent agreement (**Figure S6**).

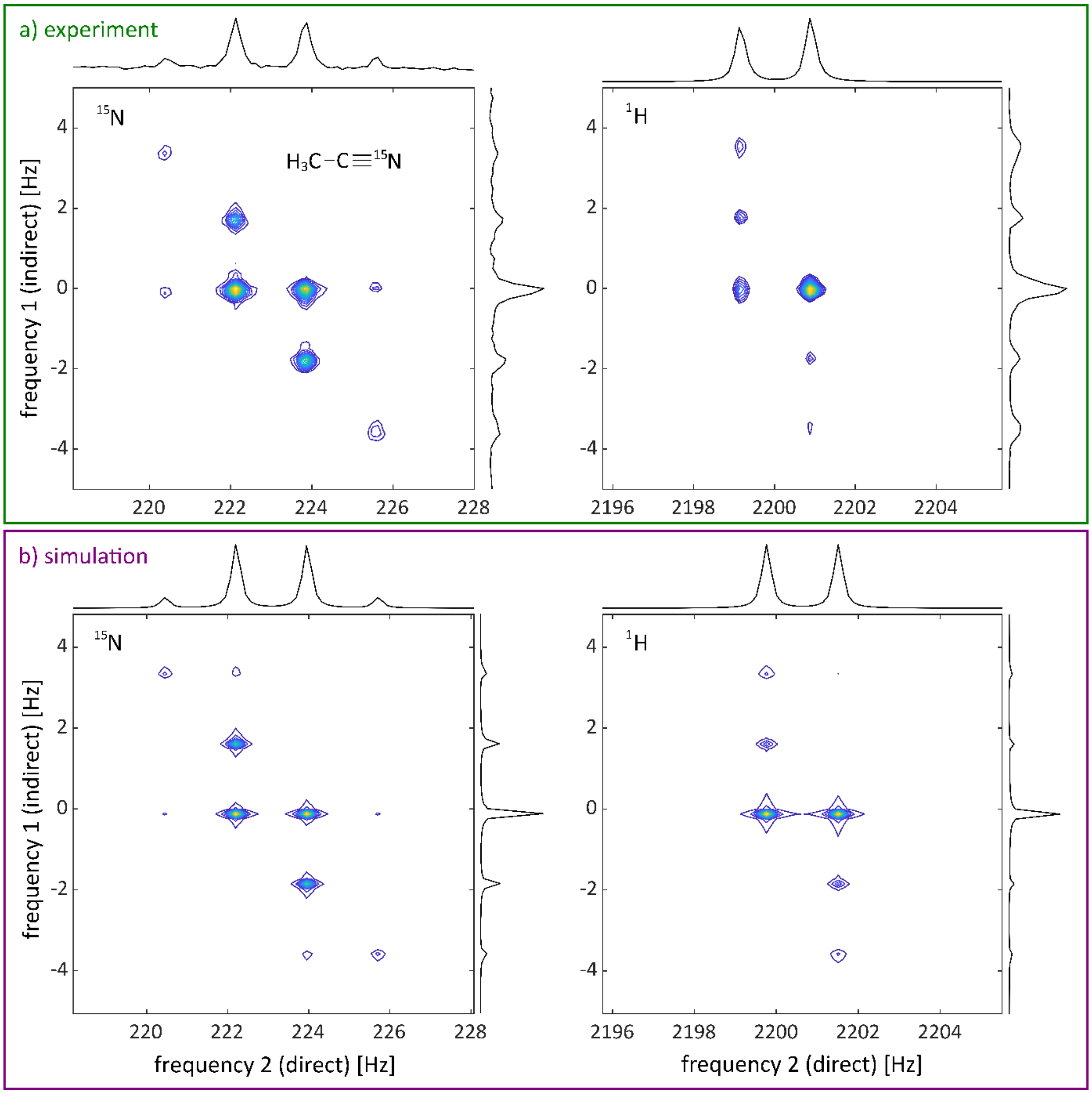


**Figure S6: ZULF COSY spectra of [$^{15}$N]acetonitrile at $B_{\mathrm{evo}}$ < 2 nT.** In (**a**) the measured data is shown, and (**b**), show simulations. The left side shows the absolute value of the $^{15}$N signal, the right side shows the absolute value of the $^{1}$H signal. In the direct dimension projection (1D spectrum above graphs), peaks at the typical quadruplet positions of $^{15}$N and doublet positions of $^{1}$H can be seen. In the indirect frequency dimension projection (1D spectrum on the right of graphs), peaks at 0, *J,* and 2*J* frequencies can be located.

## 4.2. Zeeman regime two-field COSY spectrum of [$^{15}$N]acetonitrile

The magnetic field strength of the evolution field $B_{\mathrm{evo}} = 1.3\ \mu\mathrm{T}$ falls within a range where the two-field COSY spectrum displays Zeeman-dominated interaction. In this regime, the 2D spectrum exhibits symmetrical patterns, and the peaks can be attributed to direct peaks and cross-peaks of $^{15}$N. However, the direct and cross-peaks within the indirect direction are located at $^{15}$N Larmor frequencies of $B_{\mathrm{evo}}$ (5.7 Hz respectively, **Figure S7**, brown boxes). The proton peaks are only observable as direct peaks at the proton Larmor frequencies of $B_{\mathrm{acq}}$ and the proton Larmor frequencies of $B_{\mathrm{evo}}$ (15.6 Hz). The cross-peaks of the proton are not observable. The areas where the proton peaks can be expected are marked by the blue boxes in **Figure S7**.

In the direct dimension, the spectrum reveals the anticipated quadruplet peaks for $^{15}$N, which are separated by the *J*-coupling frequency $^{3}J_{NH}$ = 1.75 Hz. Similarly, for $^{1}$H, a doublet structure is observed, also spaced by the *J*-coupling frequency. Similarly, as reported for the [1-$^{13}$C]pyruvate, there are high peaks at 0 Hz in the indirect dimension of the experimental $^{15}$N signal (**Figure S7a**). During the evolution time between the two 90° pulses, $B_{evo}$ is set to 1.3 µT, which is close to the optimal $B_{hyp}$ field of 1 µT. As a result, longitudinal polarization builds up during this period, resulting in peaks located at 0 Hz in the indirect direction. To align the simulation with experimental results, a proton polarization $p_A = 0$, and $^{15}$N polarization $p_X = 1$ were selected.

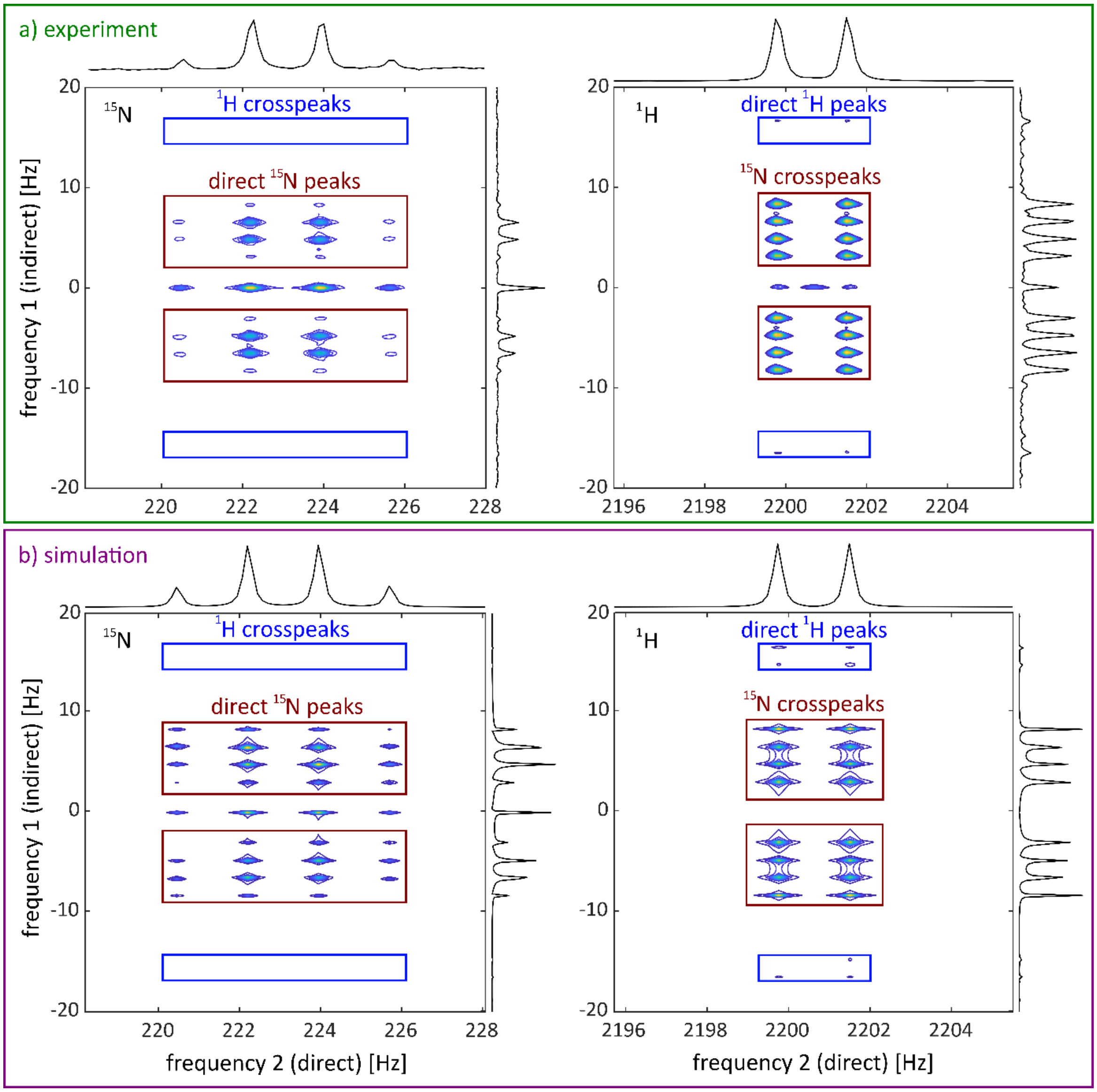


**Figure S7**: **ULF COSY spectra of [$^{15}$N]acetonitrile at $B_{evo}$ = 1305 nT.** In (**a**) the measured data is shown, and (**b**) simulations are shown. The left side shows the absolute value of the $^{15}$N signal, the right side shows the absolute value of the $^{1}$H signal. In the direct dimension projection, the spectra reveal peaks at the characteristic quadruplet positions for $^{15}$N and doublet positions for $^{1}$H.

## 4.3. ZULF regime ZULF COSY spectrum of [3-$^{19}$F]pyridine

Using [3-$^{19}$F]pyridine as a ligand in the ZULF COSY experiment yields a complex spectrum (**Figure S8**). It is observable that the spectrum has similar characteristics as the [$^{15}$N]acetonitrile and [1-$^{13}$C]pyruvate spectra, e.g., the asymmetric pattern and the indirect dimension projection being placed around 0 Hz. The peak positions also match the predicted positions from the simulations. However, the SNR is much smaller compared to the other two measured substrates, and the spectrum is far too complex to make further assumptions due to the complex $J_{FH}$ coupling pattern (2 different $^{3}J_{FH}$, as well as $^{4}J_{FH}$ and $^{5}J_{FH}$).[60] Again, there is a high orthohydrogen peak at the symmetry center of the pattern in the experimental data of the $^{1}$H data (**Figure S8a**, red box). To align the simulation with experimental results, a proton polarization $p_A = 0.29$, and $^{19}$F polarization $p_X = 0.71$ were selected. Simulation data obtained with these settings almost perfectly aligns with the experimental data.

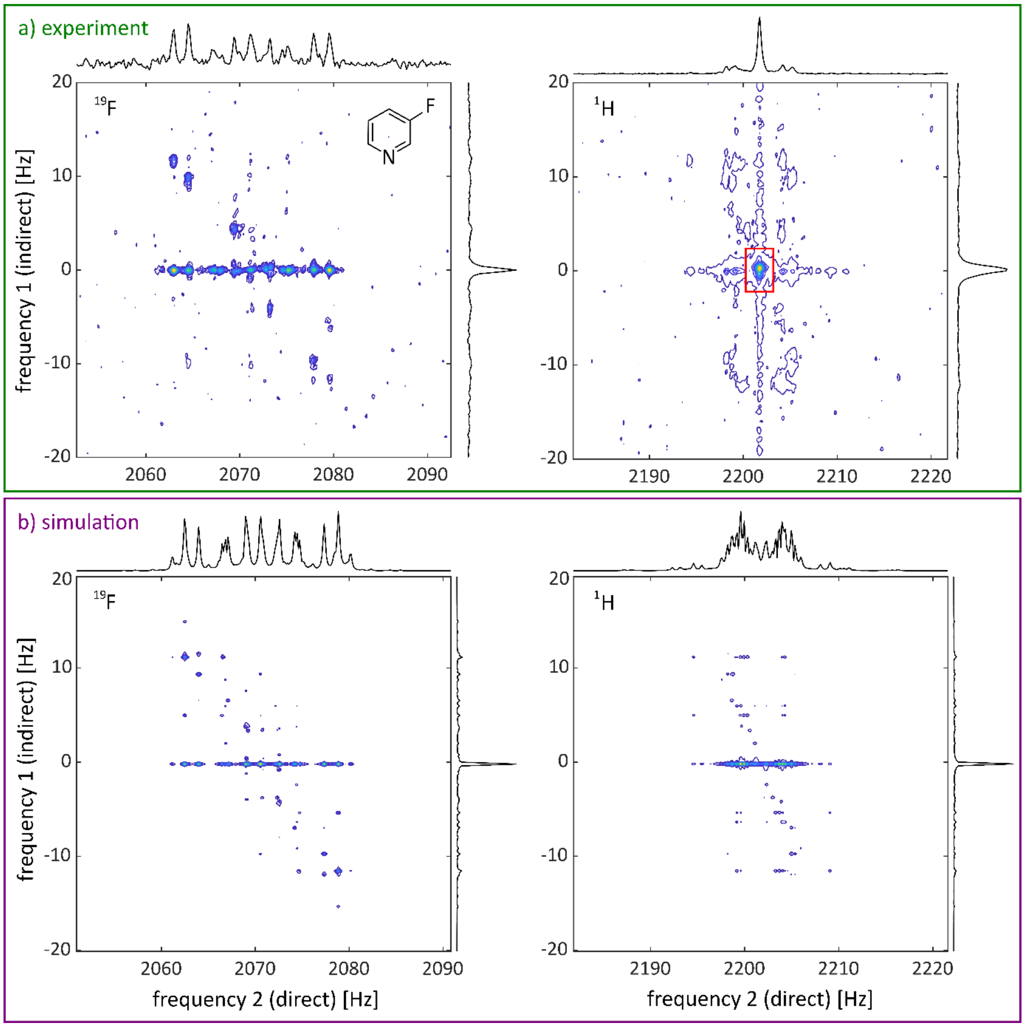


**Figure S8: ZULF COSY spectra of [3-$^{19}$F]-pyridine at $B_{evo}$ < 2 nT.** In (**a**) the measured data is shown and (**b**) show simulations. The left side shows the absolute value of the $^{19}$F signal, the right side shows the absolute value of the $^{1}$H signal. In the direct dimension projection (1D spectrum above graphs), peaks at the typical nine positions of $^{19}$F and doublet positions of $^{1}$H can be observed.

# 5. Effect of apodization on [$^{15}$N]acetonitrile and [3-$^{19}$F]pyridine ZULF COSY spectra

The effect on the unfiltered data of [$^{15}$N]acetonitrile and [3-$^{19}$F]pyridine are presented to show the robustness of the apodization formalism and function. Since the signal of [$^{15}$N]acetonitrile (**Figure S9**) is much lower than the signal of [1-$^{13}$C]pyruvate the effect of the apodization is even more observable. Applying the apodization on the [3-$^{19}$F]pyridine data (**Figure S10**), where the SNR is even lower than for [$^{15}$N]acetonitrile leads to line broadening and a reduction of resolution due to the high exponential weighting factor $k = 6$. This effect can also be observed for the [$^{15}$N]acetonitrile. The positive effect of the apodization is the improvement of the SNR and reduction of ringing, which are observable in both figures.

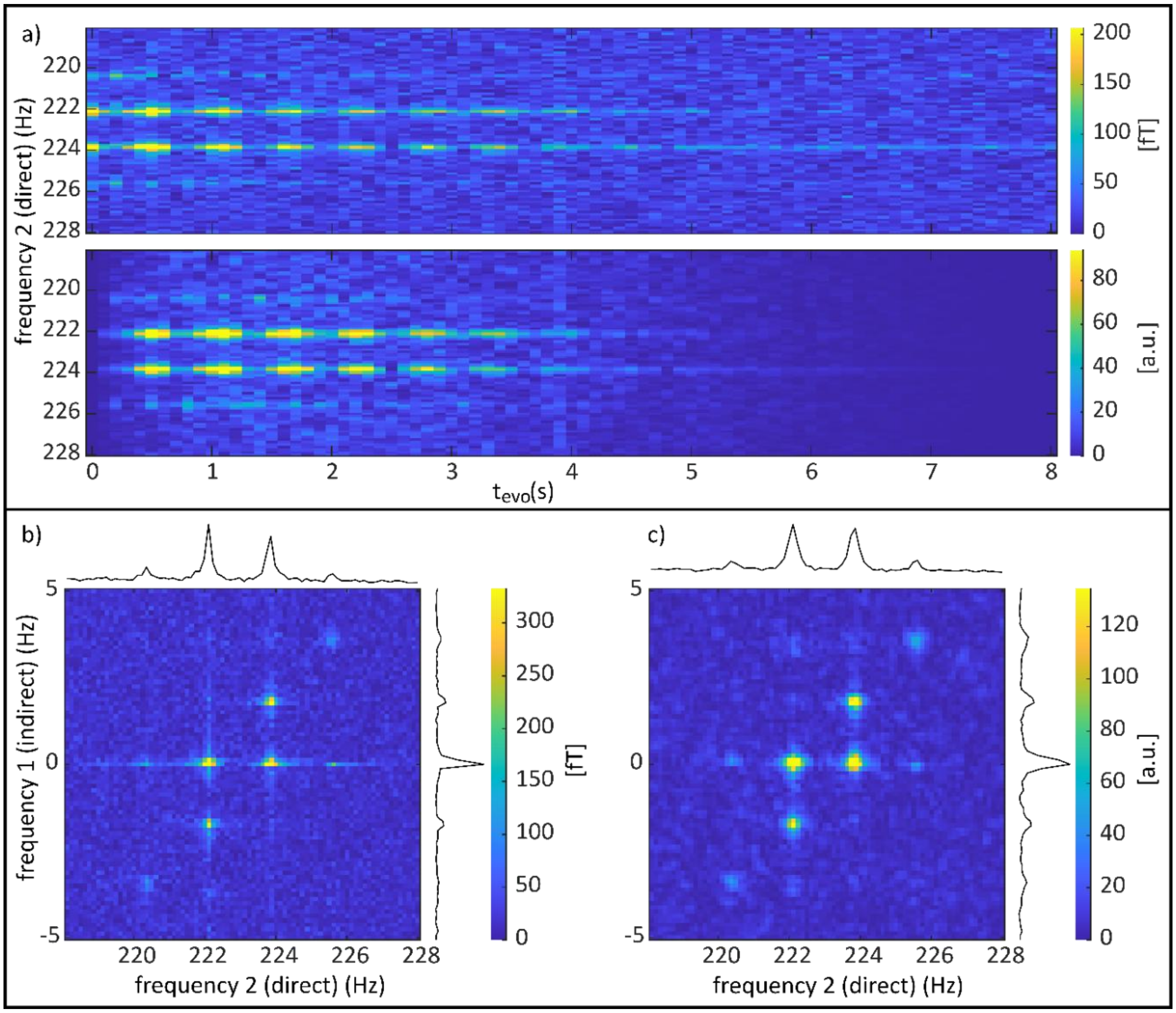


**Figure S9: Effect of the apodization on [$^{15}$N]acetonitrile data.** The figure shows the difference between the spectra with and without applied apodization. In (**a**) the direct dimension frequency dependent on the evolution time $t_{\mathrm{evo}}$ is shown. The upper figure shows the spectrum without apodization applied, while the bottom figure shows the apodized spectrum. In (**b**), the ZULF COSY spectrum in the ZULF regime, and in (**c**), the spectrum with applied apodization are displayed.

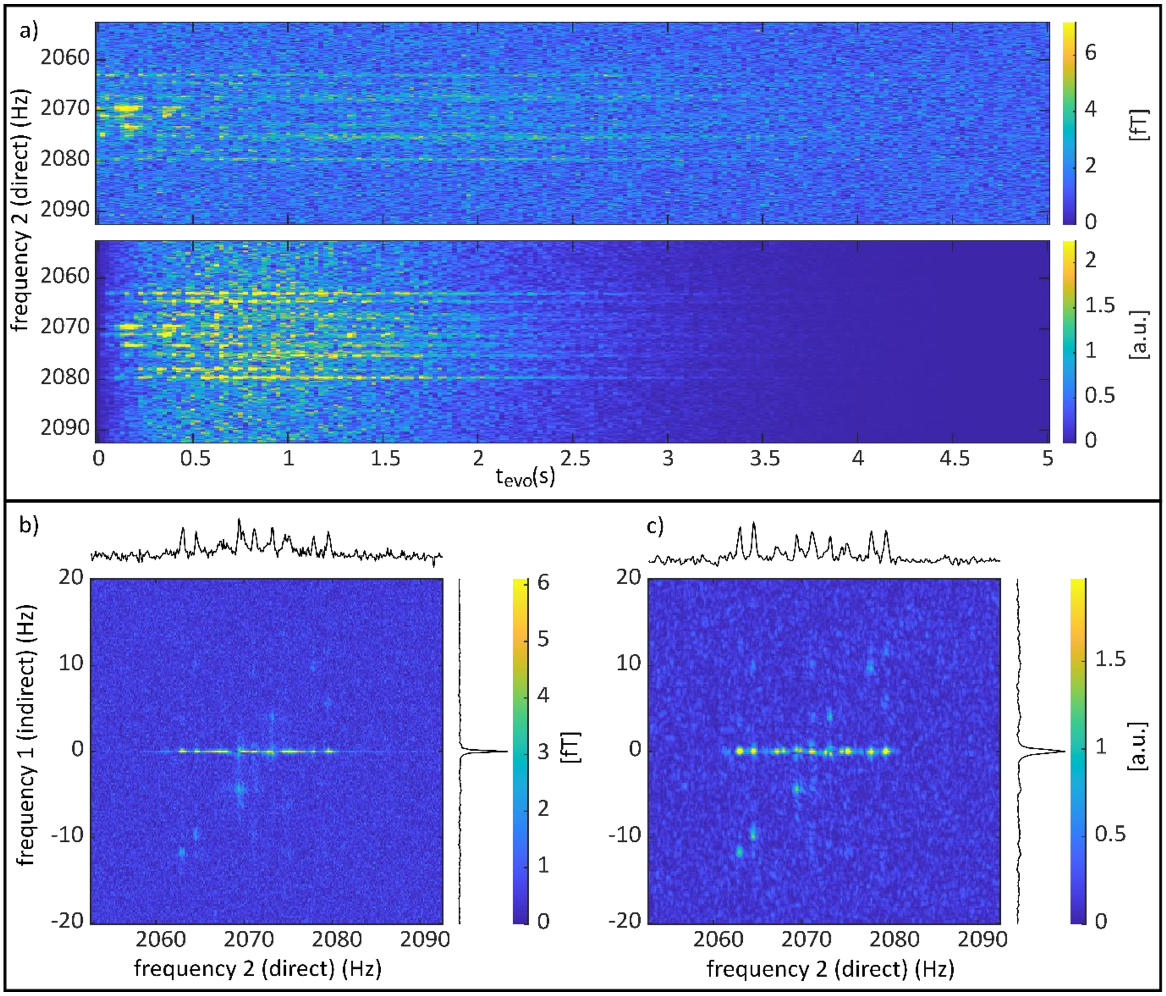


**Figure S10: Effect of the apodization on [3-$^{19}$F]-pyridine data.** The figure shows the difference between the spectra with and without apodization being applied. In (**a**) the direct dimension frequency dependent on the evolution time $t_{\mathrm{evo}}$ is presented. The upper figure shows the spectrum without apodization applied, while the bottom figure shows the apodized spectrum. In (**b**), the ZULF COSY spectrum in the ZULF regime, and in (**c**), a spectrum measured under the same condition but with applied apodization is presented.

# 6. Simulation parameters

Quantum coherences up to the third order for [1-$^{13}$C]pyruvate and [$^{15}$N]acetonitrile were populated to obtain the optimal results in the simulation. For [3-$^{19}$F]pyridine, only the first-order quantum coherences were populated. The flip angle also had to be adjusted. The simulation parameters used are shown in **Table S3**.

***Table S3: Simulation parameters.*** *The table shows the simulation parameters of the discussed results.*

| Figure | Flip angle [°] | $p_{\mathrm{A}}$ | $p_X$ | $p_{2z}$ | $p_{3z}$ | $p_{4z}$ |
|---|---|---|---|---|---|---|
| **2** | 60 | 0.09 | 0.23 | 0 | -0.68 | 0 |
| **3** | 100 | 0.074 | 0.926 | 0 | 0 | 0 |

| | | | | | | |
|---|---|---|---|---|---|---|
| **4** | 100 | 0 | 1 | 0 | 0 | 0 |
| **S6** | 60 | 0.09 | 0.23 | 0 | -0.68 | 0 |
| **S7** | 100 | 0 | 1 | 0 | 0 | 0 |
| **S8** | 60 | 0.29 | 0.71 | 0 | 0 | 0 |


(72) Lindon, J. C.; Ferrige, A. G. Digitisation and Data Processing in Fourier Transform NMR. *Prog. Nucl. Magn. Reson. Spect.*. **1980**, *14*, 27. https://doi.org/10.1016/0079-6565(80)80002-1.

(73) Benn, R.; Günther, H. Moderne Pulsfolgen in Der Hochauflösenden NMR-Spektroskopie. *Angew. Chem.* **1983**, *95*, 381. https://doi.org/10.1002/ange.19830950505.

(60) Buckenmaier, K.; Rudolph, M.; Back, C.; Misztal, T.; Bommerich, U.; Fehling, P.; Koelle, D.; Kleiner, R.; Mayer, H. A.; Scheffler, K.; Bernarding, J.; Plaumann, M. SQUID-Based Detection of Ultra-Low-Field Multinuclear NMR of Substances Hyperpolarized Using Signal Amplification by Reversible Exchange. *Sci. Rep.* **2017**, *7*, 13431. https://doi.org/10.1038/s41598-017-13757-7.
